# The toxins of vertically transmitted *Spiroplasma*

**DOI:** 10.3389/fmicb.2023.1148263

**Published:** 2023-05-18

**Authors:** Logan D. Moore, Matthew J. Ballinger

**Affiliations:** Department of Biological Sciences, Mississippi State University, Mississippi State, MS, United States

**Keywords:** *Spiroplasma*, Ixodetis, ETX/MTX2, OTU-like cysteine protease, ribosome-inactivating proteins, ankyrin, vertical transmission

## Abstract

Vertically transmitted (VT) microbial symbionts play a vital role in the evolution of their insect hosts. A longstanding question in symbiont research is what genes help promote long-term stability of vertically transmitted lifestyles. Symbiont success in insect hosts is due in part to expression of beneficial or manipulative phenotypes that favor symbiont persistence in host populations. In *Spiroplasma*, these phenotypes have been linked to toxin and virulence domains among a few related strains. However, these domains also appear frequently in phylogenetically distant *Spiroplasma,* and little is known about their distribution across the *Spiroplasma* genus. In this study, we present the complete genome sequence of the *Spiroplasma* symbiont of *Drosophila atripex*, a non-manipulating member of the Ixodetis clade of *Spiroplasma*, for which genomic data are still limited. We perform a genus-wide comparative analysis of toxin domains implicated in defensive and reproductive phenotypes. From 12 VT and 31 non-VT *Spiroplasma* genomes, ribosome-inactivating proteins (RIPs), OTU-like cysteine proteases (OTUs), ankyrins, and ETX/MTX2 domains show high propensity for VT *Spiroplasma* compared to non-VT *Spiroplasma*. Specifically, OTU and ankyrin domains can be found only in VT-*Spiroplasma*, and RIP domains are found in all VT *Spiroplasma* and three non-VT *Spiroplasma*. These domains are frequently associated with *Spiroplasma* plasmids, suggesting a possible mechanism for dispersal and maintenance among heritable strains. Searching insect genome assemblies available on public databases uncovered uncharacterized *Spiroplasma* genomes from which we identified several *spaid*-like genes encoding RIP, OTU, and ankyrin domains, suggesting functional interactions among those domain types. Our results suggest a conserved core of symbiont domains play an important role in the evolution and persistence of VT *Spiroplasma* in insects.

## Introduction

Gene duplications, losses, and horizontal transfers can facilitate dramatic shifts in bacterial lifestyle and capabilities (Romero and Palacios, 1997; Moran, 2002; Arnold et al., 2022). Gene loss is a dominant feature of symbiont evolution due to the selective benefits of removing metabolically costly genes (McCutcheon and Moran, 2012; McCutcheon et al., 2019). Alternatively, gene gain via horizontal transfer and duplication can lead to rapid adaptation across symbiotic species. For example, several mutualistic soil bacteria with nitrogen-fixing capabilities have benefited from receiving “symbiont islands” and plasmids enriched with nitrogen-fixing genes (Sullivan and Ronson, 1998; Andrews et al., 2018). Vertically transmitted symbionts are no exception to this phenomenon and can acquire novel phenotypes that benefit a heritable lifestyle via the transfer of symbiosis-supporting genes (Ballinger et al., 2019; Manzano-Marin et al., 2020; Waterworth et al., 2020).

Vertically transmitted microbial symbionts can strongly influence the life history traits of their insect hosts to maintain stable vertical transmission (VT) (Moran et al., 2008). For example, the facultative endosymbiont *Wolbachia* can defend against viral infections in their *Drosophila* hosts (Teixeira et al., 2008) but can also be penetrant reproductive parasites of *Drosophila*, using manipulative phenotypes like male-killing and cytoplasmic incompatibility to favor their transmission to subsequent generations. Heritable symbionts are ubiquitous among insects (Moran et al., 2008), and yet, a central question remains for many heritable microbes—what gene effectors promote vertical transmission and how do they arise?

*Spiroplasma* is a genus of helical, cell wall-less bacteria estimated to infect up to 7% of terrestrial arthropod species (Duron et al., 2008). The genus is phylogenetically structured into three large clades—the Apis clade, the Citri-Chrysopicola-Mirum clade, and the Ixodetis clade (Gasparich et al., 2004). Despite their rich diversity, all *Spiroplasma* are non-free-living bacteria, infecting the gut or hemolymph of invertebrates and/or the phloem of plants. At ecological timescales, *Spiroplasma* infections are transmitted horizontally by ingestion or maternally via the cytoplasm of mature oocytes (Herren et al., 2013). The Apis clade contains several insect pathogens, including *Spiroplasma taiwanense* and *Spiroplasma culicicola* of mosquitoes and *Spiroplasma apis* of European honey-bees (Mouches et al., 1982; Humphery-Smith et al., 1991; Bolaños et al., 2015). No VT *Spiroplasma* have been described in the Apis clade, while both VT and non-VT *Spiroplasma* have been reported in the Citri-Chrysopicola-Mirum clade (hereafter referred to as the Citri clade) and the Ixodetis clade (Jiggins et al., 2000; Haselkorn, 2010; Ballinger et al., 2018; Binetruy et al., 2019; Vera-Ponce León et al., 2021).

Heritable *Spiroplasma* have been described across insect orders, and are well-studied in vinegar flies, butterflies, beetles, aphids and parasitoid wasps. Some members of the Citri clade defend *Drosophila* hosts against parasitic nematodes and parasitoid wasps (Xie et al., 2010; Jaenike et al., 2010bcc Haselkorn and Jaenike, 2015). Others in this clade are both male-killers and defensive symbionts of *Drosophila* (Montenegro et al., 2005; Xie et al., 2014). Reproductive manipulation is also common among Ixodetis clade *Spiroplasma*. Ixodetis *Spiroplasma* of the butterfly *Danaus chrysippus* (*sChrys*) and the ladybird beetle *Anisosticta novemdecimpunctata* are effective VT male-killers (Jiggins et al., 2000; Tinsley and Majerus, 2006) and the recently discovered *Spiroplasma* symbiont that causes cytoplasmic incompatibility in the parasitoid wasp *Lariophagus distinguendus* (*sDis*) is also an Ixodetis clade member (Pollmann et al., 2022). Cytoplasmic incompatibility is a form of reproductive manipulation whereby toxic or modified sperm of symbiont-infected fathers kills the offspring of uninfected mothers, thus driving symbiont spread through the population. As defensive symbionts, Ixodetis *Spiroplasma* have been shown to confer resistance to fungal infections in their aphid hosts (Łukasik et al., 2013). Indeed, VT strains are present in divergent *Spiroplasma* clades, yet little is known about the genetic mechanisms supporting their host-associated phenotypes beyond those of the Poulsonii group in the Citri clade. Within the Poulsonii group, symbiont toxins have been implicated in both defensive and reproductive phenotypes (Hamilton et al., 2016; Ballinger and Perlman, 2017; Harumoto and Lemaitre, 2018).

Toxic peptides are known effectors of defensive and reproductive phenotypes across symbiotic bacterial taxa (Gillespie et al., 2018; Oliver and Perlman, 2020; Massey and Newton, 2022). *Hamiltonella defensa* utilizes phage-encoded toxins to defend aphid hosts against parasitoid wasps (Degnan and Moran, 2008). *Wolbachia* symbionts employ a toxin-antitoxin or host modification system to inflict cytoplasmic incompatibility in their hosts (Beckmann et al., 2017; LePage et al., 2017; Horard et al., 2022; Kaur et al., 2022). Within the Citri-based Poulsonii group, including *Spiroplasma* strains infecting *Drosophila hydei, Drosophila neotestacea,* and *Drosophila melanogaster* (*sHyd*, *sNeo*, and MSRO respectively), defensive strains utilize ribosome-inactivating protein toxins (RIPs) to contribute to defense against parasitoid wasps and parasitic nematodes (Hamilton et al., 2016; Ballinger and Perlman, 2017). RIPs cause irreversible damage to a key enzymatic residue of ribosomes, resulting in cell death (Stirpe, 2004). RIP gene families are remarkably diverse in Citri clade *Spiroplasma*, ostensibly due to extensive horizontal transmission, duplication, loss, and recombination (Ballinger et al., 2019; Gerth et al., 2021). *Spiroplasma poulsonii* strain MSRO is a reproductive manipulator that encodes a multidomain toxin called Spaid that causes male-killing in *Drosophila* (Harumoto and Lemaitre, 2018). Spaid consists of three integral domains including ankyrin repeat domains, an OTU-like cysteine protease domain (OTU) with predicted function in deubiquitination, and a C-terminal transmembrane domain. OTUs and ankyrin domains are also associated with type V cifB-like proteins of CI-inducing *Wolbachia*, suggesting they can be adapted for different reproductive phenotypes in diverse systems (Martinez et al., 2021). While toxins have been identified to play an essential role in maintaining symbiosis in these heritable Citri clade *Spiroplasma*, far less is known about effector genes supporting the diverse Ixodetis *Spiroplasma* phenotypes. However, recent advances in genomic representation among these strains (Yeoman et al., 2019; Martin et al., 2020; Vera-Ponce León et al., 2021; Pollmann et al., 2022) facilitates an in-depth comparative analysis of toxin gene content in this enigmatic clade.

In this study, we report the genome sequence of a *Drosophila*-associated Ixodetis clade member, the *Spiroplasma* symbiont of *Drosophila atripex* (*sAtri*). This strain does not induce male-killing or cytoplasmic incompatibility in *Drosophila* (Haselkorn et al., 2009) but does provide protection against parasitoid wasps in the family Figitidae (T. Chris Amuwa, unpublished results). We identify a diverse set of toxin and virulence domains in the genome, including the same domains contributing to defense and reproductive manipulation in the Citri clade. We conducted a genus-wide survey of 43 *Spiroplasma* genomes across all three clades to characterize the distribution of toxin domains between heritable and non-heritable *Spiroplasma*. Our results uncover a striking association of RIPs, OTUs, ankyrin, and ETX/MTX2 domains with heritable strains, suggesting that these domains play an important role in promoting VT symbiosis across *Spiroplasma*.

## Results

### Genome of *sAtri*

**Figure 1:**
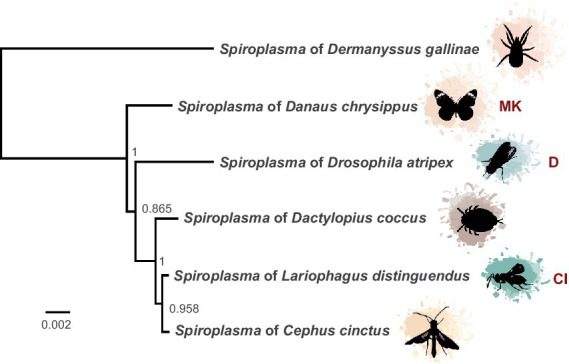
Phylogeny of heritable Ixodetis *Spiroplasma*. Midpoint-rooted phylogeny of 67 genes in ten syntenous blocks (72 kb) from available heritable Ixodetis genomes shows relationship of *sAtri* to its closest neighbors. Five gene loci from the Ixodetis *Spiroplasma* of *Dermanyssus gallinae* were used in alignment to provide additional context for phylogeny grouping. Known phenotypes are indicated with abbreviations: CI = cytoplasmic incompatibility and MK = male killing. Node labels indicate ML-like FastTree support values.

Using a hybrid long and short read sequencing strategy, we produced a closed genome assembly of 1.27 Mb for *sAtri.* The genome is covered to 29X depth by short reads (Supplementary Figure S1). Phylogenomic analysis nests *sAtri* with strong support among other Ixodetis clade endosymbiont members that infect a wide range of insect hosts (Figure 1). Genome completeness based on single copy orthologs was estimated by BUSCO at 98% (Table 1). Missing orthologs from the genome completeness analysis include the genes *rplL* and *rsmL*. We identified *rplL* in the *sAtri* genome with a manual BLAST search but were unable to identify *rsmL*. While *rsmL* (rRNA ribose-2’-O-methyltransferase) has a conserved role in bacterial translation, it is absent from all currently available Ixodetis clade genomes (Pollmann et al., 2022). The hybrid genome assembly includes eight putative plasmids, which were assigned as such based on gene content, coverage depth, and circularity (Supplementary Tables S1, S2). Repeat content is 10.73%, but only nine transposable elements could be annotated. Of the 1,313 putative protein coding genes, 639 could be assigned a functional annotation by prokka. *sAtri* encodes complete ATP synthase and glycolysis pathways, as well as transport components for glucose, fructose, GlcNAc, glycerol, and possibly mannose. Summaries of functional predictions and carbon metabolism pathway components are shown in Supplementary Figure S2A and Supplementary Table S3. Amino acid and lipid biosynthesis pathways are incomplete or absent, as reported previously for hemolymph-dwelling *Spiroplasma* species, in both the Citri and Ixodetis clades (Paredes et al., 2015; Yeoman et al., 2019; Vera-Ponce León et al., 2021). In addition to the PTS genes for carbon import (Supplementary Figure S2B), ABC membrane proteins for transport of phosphate (*ptsABCS*), nucleosides (bmpA, *nupABC*), and micronutrients (*ecfA1, ecfA2*) could be identified. A comparative summary of membrane transport, biosynthesis, and metabolism genes in *sAtri* and two other Ixodetis clade genomes is present in Supplementary Table S3. *sAtri* also encodes a Type II-C CRISPR/Cas9 system which has not been previously reported in a heritable *Spiroplasma*. No matches between *sAtri* spacers and known phages or phage regions were identified. However, several of the spacers in *sAtri* are exact matches to sequences present on *sAtri* plasmids, suggesting a possible interaction with CRISPR/Cas9 (e.g., plasmid copy number suppression). *sAtri* spacers also match to non-phage genomic sequences of other Ixodetis *Spiroplasma*, though it is unclear if those sequences are associated with plasmids.

**Table 1:** Genome assembly statistics of *Spiroplasma*.

Genome	Size (bp)	Contigs	%GC	N50	CDS	tRNAs
*Spiroplasma* of *Drosophila atripex*	1,271,056	1	23.7	Closed	1,506	27
*Spiroplasma* of *Lariophagus distinguendus*^††^	1,163,832	198	24.3	14,219	1,175	27
*Spiroplasma* of *Cephus cinctus*	713,566	145	24.9	5,160	754	23
*Spiroplasma* of *Danaus chrysippus*^†^	1,745,430	12	23.7	215,399	1,782	27
*Spiroplasma* of *Dactylopius coccus* (DCF)	1,195,508	286	23.7	6,014	1,253	27
*Spiroplasma poulsonii* strain MSRO^†^^,^^‡^	1,883,005	1	26.4	Closed	2,217	31

† Male-killing strain.

†† CI-inducing strain.

‡ Citri clade.

### *sAtri* encodes a diverse set of toxins and virulence genes

**Figure 2:**
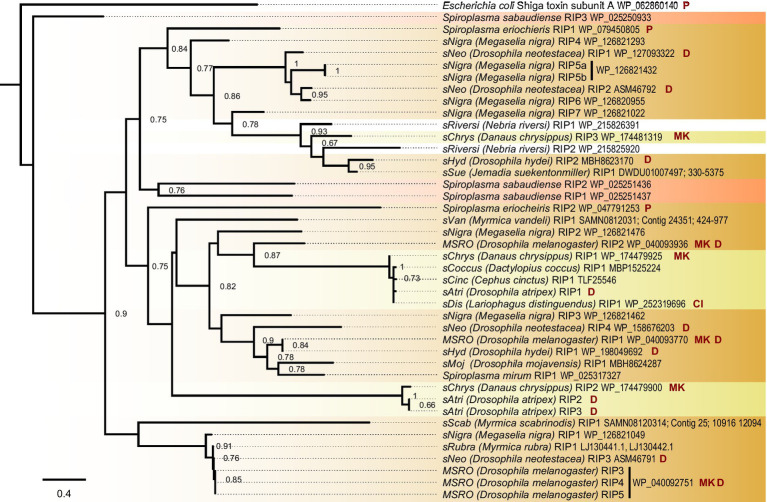
Ixodetis *Spiroplasma* encode diverse RIPs. FastTree phylogeny built from a MAFFT alignment of *Spiroplasma* RIP domain amino acid sequences and rooted to *Escherichia coli* shiga toxin subunit A. Ixodetis clade *Spiroplasma* possess multiple RIP-domain bearing proteins with a distribution explained by gains, losses, duplications, and horizontal transfers. Red shading indicates Apis clade, orange shading indicates Citri clade and yellow shading indicates Ixodetis clade. Abbreviations indicate known phenotypes of the strain from which each domain was extracted: (P: pathogenic, MK: male-killing, CI: cytoplasmic incompatibility-inducing, D: defensive). *sAtri* RIP domains are highlighted in bold. Support values greater than or equal to 0.60 are shown.

The *sAtri* genome assembly reveals similarities to and departures from other *Spiroplasma* with regard to toxin and virulence gene content. Three RIP domain-containing proteins are present, two of which encode a signal peptide for secretion. RIP toxins contribute to defense against natural enemies in some Citri clade members (Hamilton et al., 2016; Ballinger and Perlman, 2017). The *sAtri* RIPs are phylogenetically distant from one another (Figure 2) and from other non-Ixodetis *Spiroplasma* RIPs with less than 30% similarity at the amino acid level. We also identified RIPs in the genome assemblies of the *Spiroplasma* symbiont of *Danaus chrysippus* (*sChrys*) and the *Spiroplasma* symbiont of *Cephus cinctus* (*sCinc*), and retrieved the RIPs reported in the *Spiroplasma* symbiont of *Lariophagus distinguendus* (*sDis*) and the *Spiroplasma* symbiont of *Dactylopius coccus* (*sCoccus*). Examining the phylogenetic relationships of Ixodetis clade RIPs reveals them to be polyphyletic, grouping separately with distinct clades of Citri RIPs. This is consistent with ancient ancestry or horizontal transfer within the genus (Ballinger et al., 2019). *sAtri* encodes two ETX/MTX2 toxins, both of which possess signal peptides for secretion. ETX/MTX2 toxins are β pore-forming toxins that inflict cytocidal activity against their target cells and are noted for their insecticidal activity (Palma et al., 2014; Moar et al., 2017). Like *sAtri* RIPs, these toxins are diverse, grouping separately with other Ixodetis clade-encoded ETX/MTX2 toxins (Supplementary Figure S3). ETX/MTX2 toxins are numerous and widespread in *Spiroplasma* but their potential role in host-symbiont relationships is unknown. *sAtri* encodes 11 total genes containing ankyrin domains (Supplementary Figure S4) and a separate gene with an OTU-like cysteine protease. While not inherently toxic, ankyrin and OTU domains are associated with genes involved in host manipulation, and work in conjunction to cause male-killing by MSRO and possibly cytoplasmic incompatibility by *Wolbachia* (Pan et al., 2008; Nguyen et al., 2014; Harumoto and Lemaitre, 2018; Martinez et al., 2021). The OTU of *sAtri* is highly conserved among other heritable Ixodetis members (Supplementary Figure S5) and does not share an open reading frame with ankyrin domains. Despite the presence of these domains, no sex-ratio distortion has been observed in previous studies (Haselkorn et al., 2009) or in our lab stock (T. Chris Amuwa, unpublished results).

*sAtri* also encodes virulence genes that are scarcely found or not yet described in the *Spiroplasma* genus. These include an adenylate cyclase domain annotated by HMMER as anthrax toxin LF subunit. This domain is also found on the edema factor of *Bacillus anthracis* anthrax toxin. The edema factor is itself an adenylate cyclase toxin that causes large increases in the ubiquitous signaling molecule cAMP, resulting in severe disruptions to cellular processes (Tang and Guo, 2009).

*sAtri* encodes two metalloproteases of the M60 family which are implicated in virulence and infection outcomes in some bacteria and viruses (Nakjang et al., 2012; Belousov et al., 2018). M60-like peptidases, also commonly referred to as enhancins, are well studied in, but not restricted to, insect pathogenic dsDNA viruses in the family Baculoviridae and function by binding to and degrading mucin layers that protect epithelial tissues (Ishimwe et al., 2015).

### A conserved core of toxin domains in heritable *Spiroplasma*

**Figure 3:**
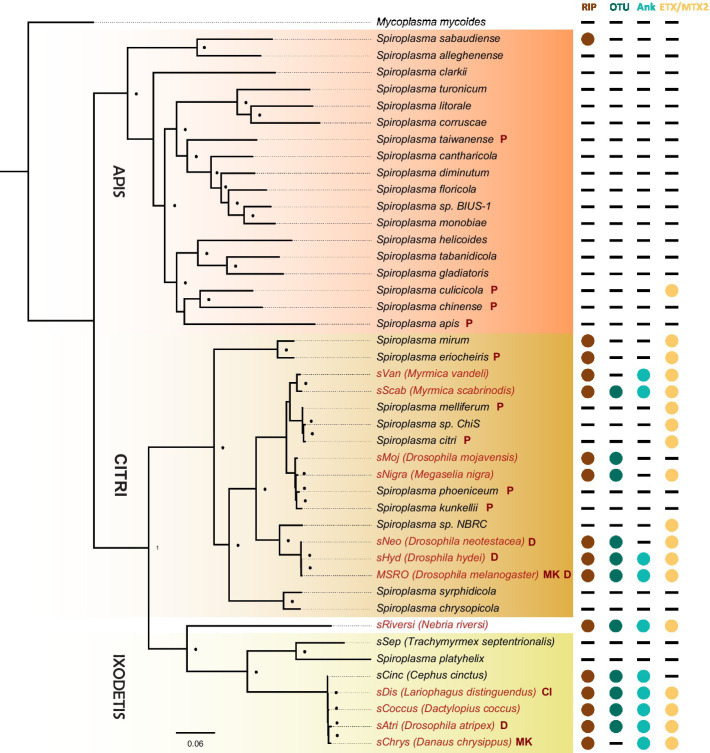
Phylogenetic distribution of select toxins and virulence domains in the genus *Spiroplasma*. FastTree phylogeny built from MAFFT nucleotide alignments of concatenated *Spiroplasma* ftsZ, rpoB and gyrB and rooted to *M. mycoides*. RIP, OTU, ankyrin, and ETX/MTX domains are preferentially distributed among VT *Spiroplasma*. Small black dots next to nodes indicate FastTree support values greater than 75%. Large colored circles to the right of branch labels indicate at least one domain copy is present in the genome. Black dash indicates no domain copies present in the genome. Red text is used for VT *Spiroplasma* and black text is used for non-VT *Spiroplasma*. Abbreviations indicate known phenotypes of the strain from which each domain was extracted: (P: pathogenic, MK: male-killing, CI: cytoplasmic incompatibility-inducing, D: defensive).

The discovery that the *Drosophila* symbiont *sAtri* harbors multiple functional domains previously reported in distantly related heritable Citri clade symbionts motivated a more detailed analysis of domain distributions across the genus *Spiroplasma*. We focused the scope of this analysis on RIP, OTU and ankyrin domains due to their role in defensive and reproductive manipulation phenotypes. We also analyzed ETX/MTX2 domains across *Spiroplasma* which are found widespread across this genus with little understanding of their role. VT-capable strains were determined based on a number of qualifying characteristics including evidence of transovarial transmission, PCR detection in hemolymph, ovaries and/or eggs, and presence of reproductive manipulation phenotypes (Table 2). VT *Spiroplasma* strains investigated in this study and their associated hosts, taxonomy and phenotypes are listed in Table 3. OTU and ankyrin domains can be found only in VT *Spiroplasma* across the Citri and Ixodetis clade (Figure 3). RIP domains are found in all VT *Spiroplasma* and in three non-VT *Spiroplasma*. ETX/MTX2 domains are more widely distributed among members of the Citri and Ixodetis clades, including most VT *Spiroplasma* and a few non-VT *Spiroplasma*. All four domains are almost entirely absent from the Apis clade except for three RIPs in *Spiroplasma sabaudiense* and a single ETX/MTX2 copy in *Spiroplasma culicicola*. Interestingly, no VT strains have been described in the Apis clade (Bolaños et al., 2015). We used BayesTraits software to identify correlations between distributions of toxin domains and heritability under two different models—one that assumes independent evolution and one that assumes dependent evolution. We find that all four domains are significantly better represented under a dependent model of evolution compared to an independent model (Table 4). For comparison, we used the same approach to determine if a correlation existed between heritability and other putative virulent *Spiroplasma* genes including *glpO* (glycerol 3 phosphate oxidase), *chiA* (chitinase), and *spi* (spiralin) (Alexeev et al., 2012; Chang et al., 2014; Duret et al., 2014). We find little to no support that the distribution of these domains across *Spiroplasma* are associated with heritability (Table 4; Supplementary Figure S6).

**Table 2:** Evidence of vertical transmission.

Strain (host)	Evidence of vertical transmission	References
*sAtri (Drosophila atripex)*	Transgenerational transmission PCR detection in hemolymph Genome sequenced from host ovaries	T. Chris Amuwa (unpublished results) and this study
*MSRO (Drosophila melanogaster)*	Transgenerational transmission Detection in hemolymph via transinfection experiments FISH visualization in eggs FISH visualization of ovaries Reproductive manipulation	Pool et al. (2006) and Harumoto and Lemaitre (2018)Herren et al. (2013)
*sNeo (Drosophila neotestacea)*	Transgenerational transmission Detection in hemolymph via transinfection experiments	Jaenike et al. (2010a) and Haselkorn and Jaenike (2015)
*sHyd (Drosophila hydei)*	Transgenerational transmission Detection in hemolymph via transinfection PCR detection in eggs (unpublished)	Mateos et al. (2006) and Xie et al. (2010)
*sMoj (Drosophila mojavensis)*	Transgenerational transmission	Haselkorn et al. (2013)
*sVan (Myrmica vandeli)*	PCR detection in hemolymph High infection frequency (92.3%) Host specificity in sympatry	Ballinger et al. (2018)
*sScab (Myrmica scabrinodis)*	PCR detection in hemolymph High infection frequency (91.9–97.6%) Host specificity in sympatry	Ballinger et al. (2018)
*sNigra (Megaselia nigra)*	Transgenerational transmission (unpublished) PCR detection in hemolymph (unpublished) High infection frequency (73.3–80.2%)	Ballinger et al. (2019)
*sRiversi (Nebria riversi)*	PCR detection in egg, larval and adult stages	Weng et al. (2021)
*sChrys (Danaus chrysippus)*	Transgenerational transmission Reproductive manipulation	Martin et al. (2020)
*sCoccus (Dactylopius coccus)*	PCR detection in ovaries	Vera-Ponce León et al. (2021)
*sDis (Lariophagus distinguendus)*	Transgenerational transmission FISH visualization in ovaries Reproductive manipulation	Pollmann et al. (2022)

**Table 3:** Vertically transmitted strains of *Spiroplasma* in study.

Host	*Spiroplasma* strain	Strain taxonomy	Phenotype
*Drosophila melanogaster*	*MSRO*	Citri-Poulsonii	Male-killing; defensive
*Drosophila neotestacea*	*sNeo*	Citri-Poulsonii	Defensive
*Drosophila hydei*	*sHyd*	Citri-Poulsonii	Defensive
*Myrmica vandeli*	*sVan*	Citri	–
*Myrmica scabrinodis*	*sScab*	Citri	–
*Drosophila mojavensis*	*sMoj*	Citri	–
*Megaselia nigra*	*sNigra*	Citri	–
*Nebria riversi*	*sRiversi*	Unplaced	–
*Dactylopius coccus*	*sCoccus*	Ixodetis	–
*Danaus chrysippus*	*sChrys*	Ixodetis	Male-killing
*Lariophagus distinguendus*	*sDis*	Ixodetis	Cytoplasmic incompatibility
*Drosophila atripex*	*sAtri*	Ixodetis	Defensive

Dash (−) indicates a phenotype has not yet been published.

**Table 4:** Model comparisons for describing toxin and virulence domains across heritable *Spiroplasma*.

Domain	Dependent model likelihood score	Independent model likelihood score	Chi score	df	value of p*
RIP	−29.855283	−40.389039	21.067512	4	0.000307
OTU	−26.546300	−37.764235	22.43587	4	0.000164
ankyrin	−26.036137	−35.577156	19.082038	4	0.000757
ETX/MTX2	−33.930099	−44.915624	21.97105	4	0.000203
glpO	−44.902777	−46.963108	4.056606	4	0.398347
chiA	−37.674589	−40.678499	6.00782	4	0.198552
spiralin	−32.183710	−37.138576	9.91	4	0.041971

* Value of *p* threshold has been set at 0.001.

### VT-specific domains vary in copy number and origin

**Figure 4:**
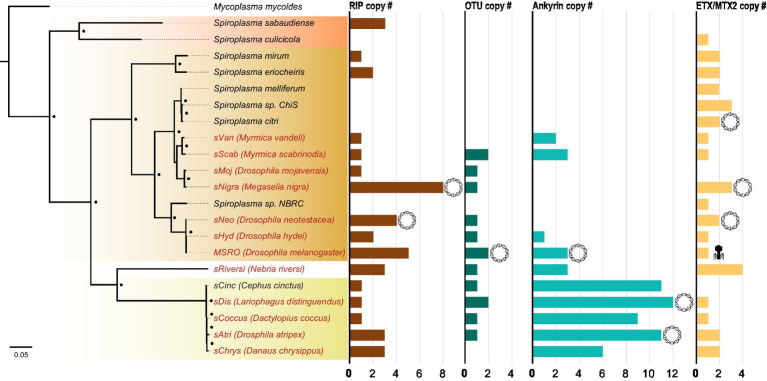
Number of toxin and virulence domains varies across *Spiroplasma* genus. FastTree phylogeny built from MAFFT nucleotide alignment of concatenated *Spiroplasma* ftsZ, rpoB, and gyrB sequences and rooted to *M. mycoides*. All *Spiroplasma* present in the phylogeny encode at least one domain copy of RIP, OTU, ankyrin, or ETX/MTX2. Bar graphs show the number of domain copies present in each genome for each domain type. Double-helix circles indicate at least one of the domains are found on a plasmid. Phage illustrations indicate domains are found within a phage region of the genome. Red shading represents Apis clade, orange shading represents Citri clade and yellow shading represents Ixodetis clade. Red color text is used for VT *Spiroplasma* and black color text is used for non-VT *Spiroplasma*. Small black dots indicate FastTree support values above 0.75.

While RIP, OTU, ankyrin, and ETX/MTX2 domains are distributed across diverse heritable *Spiroplasma*, the number of genes possessing these domains can vary drastically, even between closely related strains (Figure 4). For example, the number of genes possessing RIP domains varies both within and between clades, likely due to gene duplications and horizontal gene transfers. The number of genes possessing ankyrin domains are consistent within clade but show extreme copy number variation between clades characterized by ankyrin enrichment in Ixodetis *Spiroplasma*. Conversely, the number of OTU and ETX/MTX2 copies is relatively consistent within and between clades. All four of these domains can be found on plasmids (Harumoto and Lemaitre, 2018; Ballinger et al., 2019). In some cases, a given strain’s entire domain repertoire can only be found on plasmids such as ankyrins and OTUs in MSRO, and ETX/MTX2 in *Spiroplasma citri*. In contrast, only one domain could be found within an endogenous phage region (ETX/MTX2 of MSRO), suggesting *Spiroplasma* phages do not explain the distribution of RIP, OTU, ankyrin, or ETX/MTX2 domains across the genus.

### VT-specific domains source diverse recombinant genes

**Figure 5:**
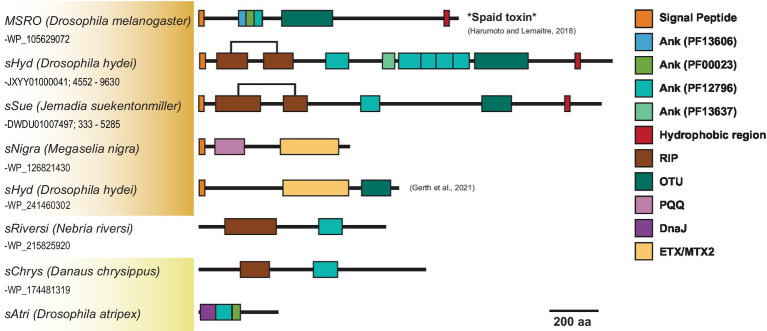
Dynamic variants of toxin and virulence genes across *Spiroplasma*. Scaled representation showing diverse array of *Spiroplasma* toxin-bearing proteins identified in this study. Orange shading indicates Citri clade and yellow shading indicates Ixodetis clade. A bracket is used for *sHyd* and *sSue* to highlight that the two separated RIP domains make up a whole RIP domain. Spaid toxin responsible for male-killing by MSRO is shown above.

Across Citri and Ixodetis-clade *Spiroplasma*, we observe multiple structural variants of RIPs, OTUs, ankyrins, and ETX/MTXs within the same open reading frame (Figure 5). In a particularly interesting case, we uncovered two Spaid-like toxins from the genome of the *Spiroplasma* endosymbiont of *Drosophila hydei* (strain *sHyd1*, NCBI BioProject PRJNA274591) and a *Spiroplasma* genome extracted from whole-genome sequence data of the South American butterfly *Jemadia suekentonmiller* (*sSue*). These open reading frames include ankyrin domains, an OTU domain, and the Spaid C-terminal transmembrane domain. Toward the N-terminus, these proteins are also equipped with two RIP domains which together possess all the conserved active site residues of a single RIP domain. These Spaid-like toxins are present across several *Spiroplasma* genomes extracted from hesperid butterfly assemblies (Supplementary Figure S7).

### *Spiroplasma* with VT-associated domains are widespread across Hesperiidae butterflies and other insects

**Figure 6:**
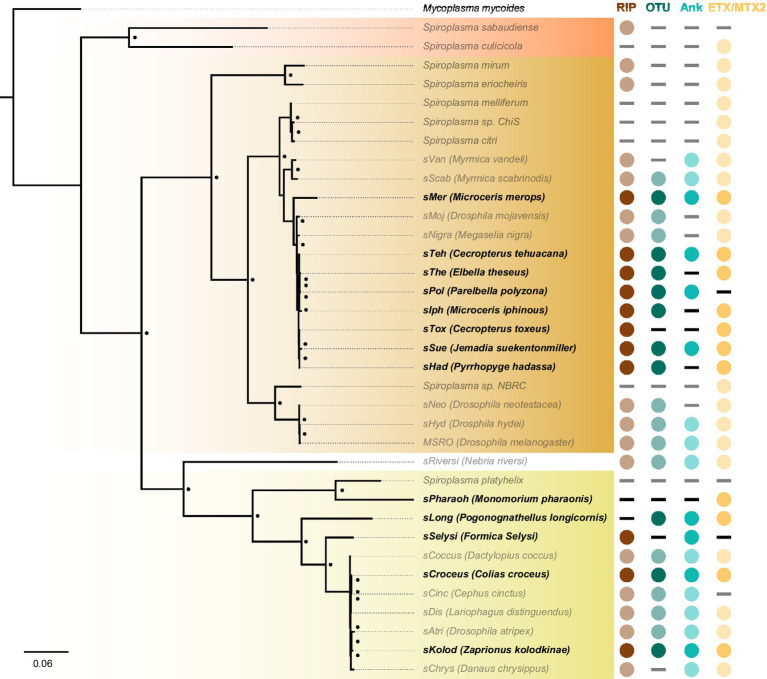
Cryptic *Spiroplasma* carry domains associated with a heritable lifestyle. FastTree phylogeny built from MAFFT nucleotide alignment of concatenated *Spiroplasma* ftsZ, rpoB, and gyrB and rooted to *M. mycoides.* This phylogram shows the placement of novel *Spiroplasma* strains (black bold) extracted from insect WGS sequences. Many of these *Spiroplasma* strains carry RIPs, OTUs, and/or ankyrin domains that are strongly associated with a heritable lifestyle. Circles indicate the presence of a given domain and dashes indicate their absence. Red shading indicates Apis clade, orange shading indicates Citri clade and yellow shading indicates Ixodetis clade. WGS-extracted taxa are shown in bold typeface. Black dots adjacent to nodes indicate a FastTree support value greater than 0.75.

We further investigated the presence of *Spiroplasma* in insect genome assemblies using MSRO Spaid, and FtsZ and rpoB proteins from Apis, Citri, and Ixodetis clade taxa as queries against whole genome shotgun (WGS) sequence databases on NCBI and identified Citri clade *Spiroplasma* genomic sequences in several South American members of the Hesperiidae butterfly family. A few of these *Spiroplasma* infections are also reported in an unpublished preprint (Medina et al., 2020; Manzano-Marin et al., 2020). These Hesperiidae-infecting *Spiroplasma* strains group closely to the heritable *Spiroplasma* strains infecting *Drosophila mojavensis* and *Megaselia nigra* (*sMoj* and *sNigra*, respectively) (Figure 6). Metagenomic binning of *Spiroplasma* contigs reveals this novel group of *Spiroplasma* is also equipped with RIPs, OTUs, ankyrins, and ETX/MTX2 domains. Phylogenetic analysis of Ixodetis clade *Spiroplasma* extracted from WGS sequences reveals an association with diverse insects including two ants, a butterfly, a springtail and a drosophilid (Figure 6). The presence of RIPs, OTUs, ankyrins, and ETX/MTX2 domains is variable among these novel Ixodetis strains (Figure 6). For example, the *Spiroplasma* genome extracted from a *Monomorium pharaonic* ant assembly (*sPharaoh*) is estimated to be 98% complete (Supplementary Table S4) and only encodes a single ETX/MTX2 domain. Alternatively, Ixodetis clade *Spiroplasma* genomes extracted from the assemblies of the butterfly *Colias croceus* (*sCroceus*) and the drosophilid *Zaprionus kolodkinae* (*sKolod*) both encode RIPs, OTUs, ankyrins, and ETX/MTX2 domains.

## Discussion

In this study, we report a conserved core of toxin and virulence domains associated with heritable *Spiroplasma*. As one of the most successful and diverse arthropod-associated heritable symbionts on Earth (Duron et al., 2008), the mechanisms that support *Spiroplasma* persistence across ecological timescales are of great interest and yet largely unknown. Previous work has demonstrated that *Spiroplasma* exploit conserved yolk protein import machinery to invade maturing oocytes, providing a possible explanation for their effective vertical transmission in diverse arthropods (Herren et al. 2013). But how *Spiroplasma* navigates numerous other obstacles required for growth and persistence in host hemolymph throughout host development is not well understood. While toxins are often viewed through the lens of pathogenesis, they can also mediate and manipulate host processes to maintain symbiont persistence. For instance, RIPs and an OTU-ankyrin-bearing Spaid toxin promote *Spiroplasma* persistence by supporting defensive and reproductive phenotypes, respectively (Hamilton et al., 2016; Ballinger and Perlman, 2017; Harumoto and Lemaitre, 2018). Our assembly of the Ixodetis *Spiroplasma* genome from *Drosophila atripex* and accompanying comparative analyses reveal that the presence of these domains is highly conserved across diverse heritable *Spiroplasma*. RIPs, OTUs and ankyrin domains are specific to VT *Spiroplasma*, and are rare (RIPs) or entirely absent (OTUs and ankyrins) in non-VT *Spiroplasma* genomes. These toxin and virulence domains may play a role in facilitating and maintaining a vertically transmissible lifestyle across heritable *Spiroplasma* as has been demonstrated for a small number of Citri clade members. Finally, ETX/MTX2 toxins are more widespread in non-VT Citri and Ixodetis *Spiroplasma* compared to RIPs, OTUs, and ankyrins but are still highly correlated with a VT lifestyle based on BayesTraits analysis. As previously stated, the role of ETX/MTX2 toxins in host-*Spiroplasma* interactions is unknown but their propensity for VT-*Spiroplasma* may suggest a role in maintaining a VT lifestyle similar to RIPs, OTUs and ankyrin domains (i.e., defense or host manipulation).

We find that similar toxin evolutionary processes previously reported in Poulsonii clade strains (Ballinger et al., 2019; Gerth et al., 2021) are mirrored in toxin domain-containing proteins of the Ixodetis clade, suggesting conservation of dominant toxin evolution mechanisms across heritable strains throughout the genus. Citri clade RIPs experience duplications, losses, and domain swapping events that have accompanied an explosion in RIP diversity. Similarly, Ixodetis RIPs are polyphyletic and RIP representation differs between strains. For example, a RIP from the Ixodetis endosymbiont of the butterfly *Danaus chrysippus* groups closely with the enigmatic double RIP domain-possessing proteins recently uncovered from *sHyd1* and *sSue* (Figure 5; Gerth et al., 2021). Ankyrin domain-containing proteins are especially numerous in members of the Ixodetis clade, ranging from six to fourteen copies across members of this clade. Believed to originate in eukaryotes, ankyrin domains are more commonly found in heritable microbes than other bacterial taxa (Jernigan and Bordenstein, 2014), and may facilitate a variety of microbe-host interactions. This makes the expansion of ankyrin domains in Ixodetis particularly interesting, especially given the broad range of insect hosts they infect (Figure 1).

Dynamic evolution is also demonstrated in the varied number of genes that possess these domains (i.e., RIP, OTU, ankyrin, and ETX/MTX2)—even among closely related strains—indicating that duplications, gains, and losses are common generators of their diversity in *Spiroplasma*. RIP, OTU, ankyrin, and ETX/MTX2 domains are also present on plasmids, possibly contributing functional roles that favor their dispersal and persistence through the genus. Plasmids often confer adaptive phenotypes to their bacterial hosts to help drive their own spread among bacterial populations (Dimitriu et al., 2021). Interestingly, no RIP, OTU, or ankyrin domains are present within the diverse plasmids of non-VT Citri clade *Spiroplasma*. For example, some *Spiroplasma citri* strains can have upwards of nine unique plasmids (Rattner et al., 2021). Given the promiscuity of plasmids across *Spiroplasma¸* the lack of RIP, OTU, and ankyrin domains on plasmids in non-heritable strains suggests that they may have little adaptive function in that lifestyle.

Not only do RIPs, OTUs, and ankyrin domains exist within diverse proteins across *Spiroplasma*, but many VT *Spiroplasma* species are equipped with several structural variants of these domains on the same protein. In one notable example, our analysis revealed a *Spiroplasma* genome within the WGS assembly of the South American butterfly *J. suekentonmiller* that encodes a protein with RIP domains, an OTU domain, an ankyrin domain and a C-terminal transmembrane domain, i.e., a RIP-Spaid fusion protein. These VT-associated domains appear to frequently recombine to create novel protein configurations. For instance, Spaid and Spaid-like proteins encode multiple ankyrin domains each and these ankyrin domains vary greatly in copy number and protein family (Figure 5; Supplementary Figure S7B), suggesting that ankyrin-spanning region is especially prone to domain losses and gains. The close association of RIPs, OTUs, and ankyrins on the same proteins across Citri and Ixodetis *Spiroplasma* suggests functional evolutionary ties between these domains.

Extracting symbiont genomes from publicly available nucleotide databases is a powerful and convenient approach to studying symbiont evolution (Salzberg et al., 2005; Gerth and Hurst, 2017; Pascar and Chandler, 2018; Scholz et al., 2020; Pilgrim et al., 2021). We explored the presence of RIP, OTU, ankyrin, and ETX/MTX2 domains in other insect assemblies by mining partial to near complete genomes of both Ixodetis and Citri clade *Spiroplasma* from a diverse array of insect WGS assemblies. Within Citri, there is a large group of *Spiroplasma* strains infecting various members of the South American butterfly family Hesperiidae, and are equipped with RIP, OTU, and ankyrin domain-possessing proteins. Within Ixodetis, we identified five novel *Spiroplasma* genomes extracted from a diverse variety of insect hosts with a varied presence of RIP, OTU, ankyrin, and ETX/MTX2 domains. This analysis provides promising candidates for future studies on heritable *Spiroplasma* and the identification of RIP, OTU, ankyrin, and ETX/MTX2 domains within insect WGS assemblies may be useful for developing early hypotheses on *Spiroplasma*-host interactions.

## Methods

### Genome sequencing, assembly, and annotation

Nucleic acids were extracted from ovaries of eight adult female *Drosophila atripex* using the phenol-chloroform method. Paired end 150 bp Illumina genomicDNA reads were sequenced by Novogene (CA, United States). Long reads were sequenced on an Oxford Nanopore MinION device and R9.4.1 flow cell following library preparation using the Ligation Sequencing Kit (SQK-LSK109). Guppy 6.1.5 implemented in MinKNOW 22.05.5 was used to generate high accuracy basecalls. Only long reads above 10 kb were used in hybrid assembly. Illumina reads were trimmed for adapter sequences and quality (qtrim = r trimq = 10) with BBMap 38.35 (Bushnell, 2014), and a metagenome was hybrid assembled using Unicycler 0.5.0 (Wick et al., 2017). *Spiroplasma*-derived contigs were retained through a combination of metagenomic binning (Laczny et al., 2017) and manual filtering. Manual filtering was performed through targeted searches of sequences likely to contaminate the bin based on similar nucleotide content and was guided by kmer abundance analyses performed with KAT (Mapleson et al., 2016). The draft genome was annotated through Prokka 1.14.5 (Seemann, 2014) and KEGG numbers were assigned with BLASTKoala (Kanehisa et al., 2016). Genome coverage and plasmid coverage were determined using BBMAP (Bushnell, 2014). Genome completeness was estimated with BUSCO v5 (Simão et al., 2015) on the gVolante webserver (Nishimura et al., 2017). *Spiroplasma* homologs of interest, including those used for phylogenetics, were identified using HMMER (Finn et al., 2011) and PfamScan (Mistry et al., 2021) with a threshold of E-value = 0.01. Proteins of interest that could not be annotated by the previously mentioned approaches were investigated further with HHpred (Zimmermann et al., 2018), BLASTp and alignments. Protein and nucleotide sequences were aligned with MAFFT 7.388 (Katoh and Standley, 2013) and phylogenies were built using FastTree 2.1.11 (Price et al., 2010) unless stated otherwise. BLASTp searches with relaxed significance thresholds (expect values ≤0.01) were also performed to ensure detection of more distantly related protein domains if present.

### Characterizing distribution of heritability, toxin, and virulence domains across *Spiroplasma* genus

All *Spiroplasma* sp. genomes, plasmids, and reads were downloaded from NCBI, and their accession numbers are listed in Supplementary Table S5. Heritable *Spiroplasma* were identified based on evidence provided from transgenerational screenings, ovarian tissue and egg screens, high infection frequencies among populations, multi-year screenings and systemic infections (Table 2). We searched for RIP, OTU, ankyrin, and ETX/MTX2 domains in all *Spiroplasma* genomes using tBLASTn (threshold E-value = 0.01) with a curated list of *Spiroplasma* domains that have been identified in this study and extracted using HMMER domain annotations as a guide (Supplementary Table S6). Protein domains were confirmed with HMMER 3.3 and pfamscan with a.01 e-value threshold, and through alignments to confirm presence of conserved residues. Domains present on open reading frames and on pseudogenes are both included in the total count for domains of interest. Phage regions were determined using Phaster webserver (Zhou et al., 2011; Arndt et al., 2016). Annotated phage regions were extracted from the genomes of *Spiroplasma* strains known to contain RIP, ankyrin, OTU, or ETX/MTX2 domains. These phage regions were investigated to determine if they included any of these domains.

We determined whether a correlation existed between heritability and specific encoded domains using the software BayesTraits V4.0.0 (Pagel and Meade, 2006). Heritability was treated a discrete binary trait (heritable or non-heritable) and domains were also treated as a binary trait (domain present domain absent in genome). Likelihood scores were calculated for both a dependent model (i.e., assumes heritability and domain acquisition/retention evolved dependently) and an independent model (i.e., assumes heritability and domain acquisition/retention evolved independently). Likelihood scores were compared with a chi-square analysis as recommended by BayesTraits documentation. We rejected the null hypothesis of independent evolution at *p* < 0.001.

### Identification and extraction of *Spiroplasma* genomes from insect whole genome shotgun assemblies

Using Spaid toxin from MSRO, and ftsZ and rpoB from Ixodetis, Citri, and Apis clade taxa as queries, we performed tBLASTn searches of whole genome shotgun contigs (WGS) located on the NCBI database. Default search parameters were used, and we limited our organism search to Insecta (taxid:50557). Assemblies with matches to these genes were submitted to BusyBee web server for genomic binning (Laczny et al., 2017). Bins outputted by BusyBee were manually inspected for the presence of core *Spiroplasma* genes including ftsZ, rpoB, and gyrB. These core genes were also used to construct the phylogeny in Figure 6. We performed tBLASTn against the extracted *Spiroplasma* genomes using a curated list of *Spiroplasma* RIP, OTU and ankyrin peptide sequences. If a domain was determined to be missing from an extracted *Spiroplasma* genome, we searched for it in the original insect assembly to ensure it wasn’t present on a *Spiroplasma* contig missed in the binning process. Genome completeness was determined using BUSCO through gVolante webserver, and coding DNA sequences and tRNA content was determined with Prokka 1.14.5. Genome statistics and insect assembly accession numbers are available in Supplementary Table S4.

### Constructing phylogenies

MAFFT 7.388 was used to create all alignments and FastTree 2.1.11 was used to construct *Spiroplasma* phylogenies. Jukes-Cantor model was used to build phylogenies from nucleotide sequence alignments. RIP and OTU proteins are often flanked with diverse, nonhomologous accessory domains. For this reason, RIP and OTU regions immediately outside of the conserved active site residues were trimmed out of alignments manually; conserved active site residues span the majority of both domains. *sSue* RIP, *sHyd* RIP2, and *sChrys* RIP3 all possess peptide insertion sequences ranging from 54 to 157 aa that split their RIP domain. This appears to be a conserved feature among this clade of RIPs. Due to their large size and highly divergent sequence variation, we identified these insertion sequence regions using alignments to other RIPs and removed them manually. *sHyd* RIP2 and *sRiversi* RIP1 also group among these RIPs, however they are small proteins encoding only a partial RIP domain and therefore did not require additional trimming. The trimmed RIP and OTU sequences were then aligned with MAFFT 7.388 and submitted to MEGA software (Kumar et al., 2018) to determine the best substitution model for PhyML tree building. WAG+G + I substitution model was used to construct a RIP phylogeny and cpREV+G substitution model was used to construct an OTU phylogeny. ETX/MTX2 are divergent toxins that lack conserved active site residues to help guide trimming. We created an alignment of ETX/MTX2-possessing proteins with MAFFT 7.388 and confirmed that the annotated ETX/MTX2 domains were aligned. The alignment was then trimmed using ClipKIT software (Steenwyk et al., 2020) in kpi-gappy mode. The trimmed alignment output was submitted to MEGA software to determine the best substitution model for PhyML tree building. WAG+G + F substitution model was used to construct an ETX/MTX2 phylogeny.

## Data availability statement

The datasets presented in this study can be found in online repositories. The names of the repository/repositories and accession number(s) can be found at: The Bioproject accession is “PRJNA928682” and the Biosample accession is “SAMN32939082” and the SRA accessions are “SRX19199603” and “SRX19199602.”

## Author contributions

LM and MB conceived and designed the study, collected data and performed analyses, and revised the manuscript and approved the final submission. LM prepared figures and wrote the first draft of the manuscript. All authors contributed to the article and approved the submitted version.

## Funding

This work was supported by National Science Foundation award 2144270 to MB.

## Conflict of interest

The authors declare that the research was conducted in the absence of any commercial or financial relationships that could be construed as a potential conflict of interest.

## Publisher’s note

All claims expressed in this article are solely those of the authors and do not necessarily represent those of their affiliated organizations, or those of the publisher, the editors and the reviewers. Any product that may be evaluated in this article, or claim that may be made by its manufacturer, is not guaranteed or endorsed by the publisher.
