# Supplementary material for: The toxins of vertically transmitted Spiroplasma
Source: Front Microbiol. 2023 May 18;14:1148263. doi: 10.3389/fmicb.2023.1148263 (PMC10232968; doi:10.3389/fmicb.2023.1148263)
Supplement: Supplementary file 1 [file Data_Sheet_1.zip › Supplementary Figures.docx]

**Supplementary figures**

| **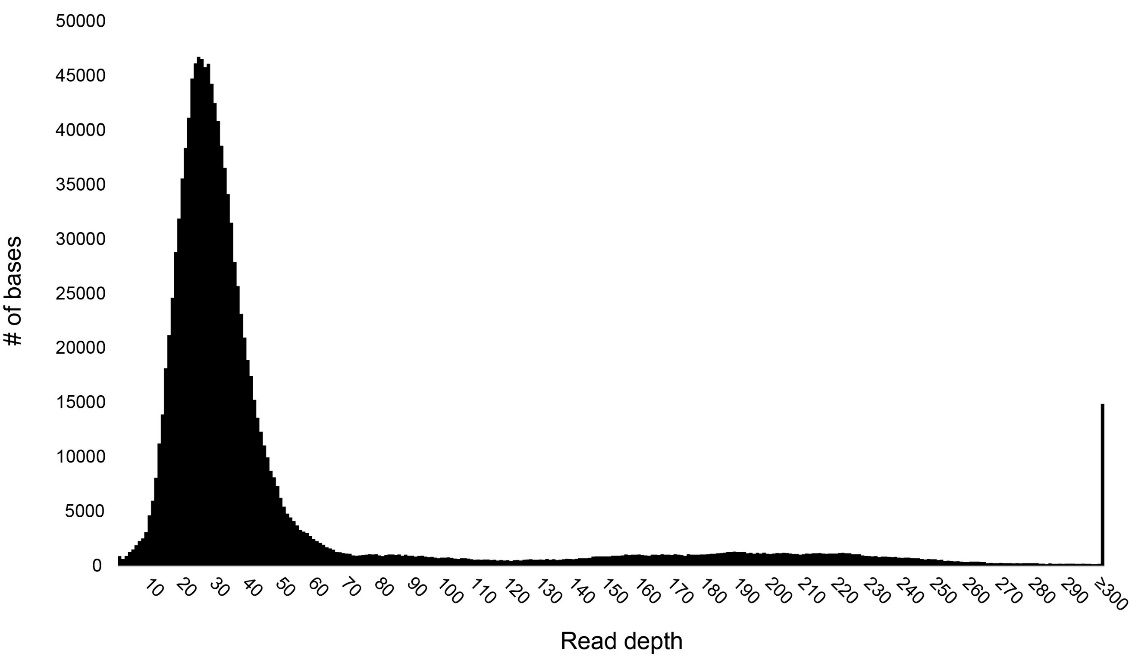** |
| --- |
| **Figure S1 *sAtri* genome read depth.** Coverage depth distribution of 125 bp Illumina HiSeq reads mapped to the *Spiroplasma*-assigned metagenomic bin of the Illumina assembly. |

| **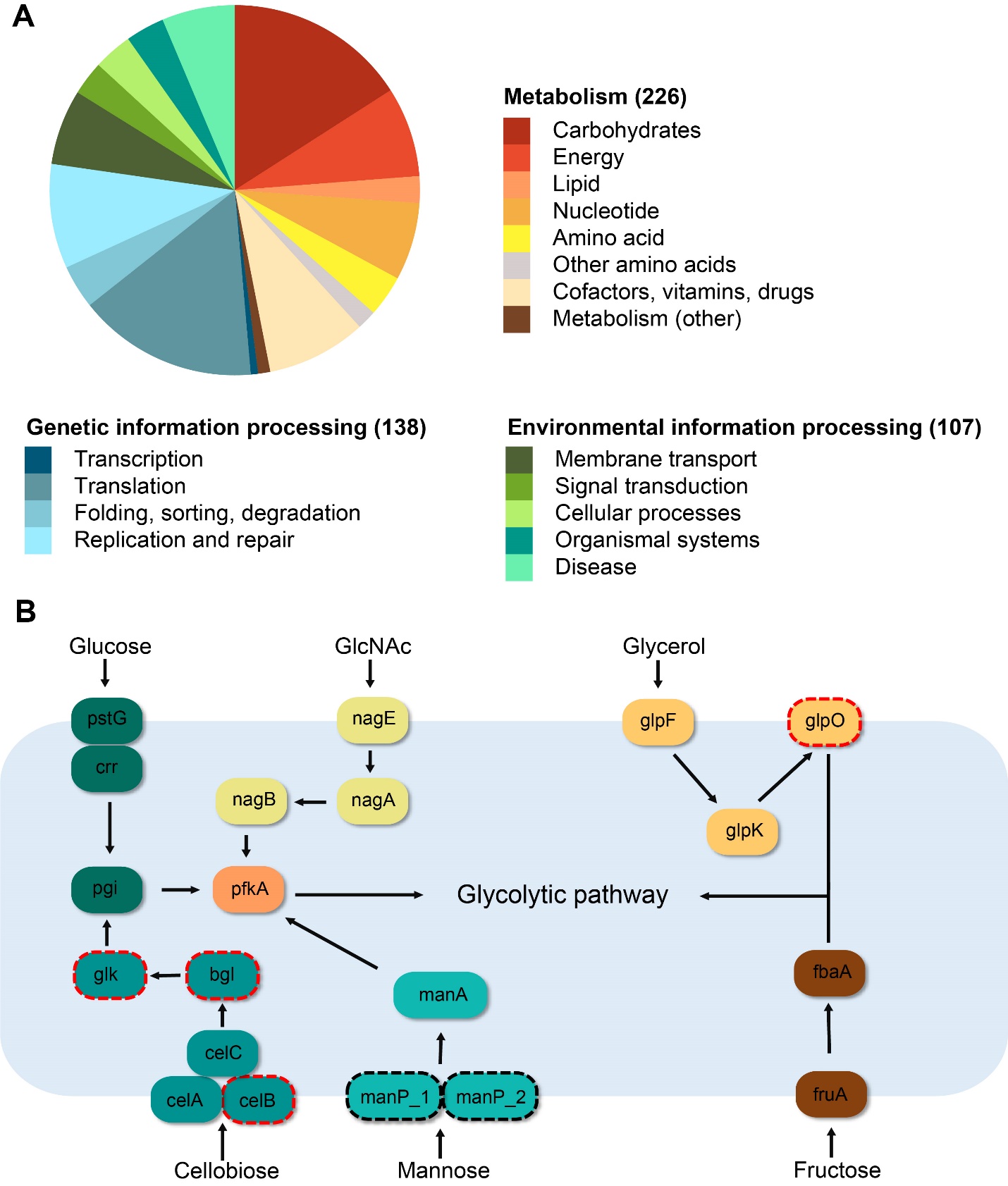** |
| --- |
| **Figure S2. *sAtri* gene content and metabolism**. A) A summary of sAtri gene content organized by function according to KEGG annotations. Numbers beside category headings indicate the number of genes. B) A summary of *sAtri* metabolic pathways and transporters. Red-dotted outline indicates gene is missing or it is a pseudogene. Black-dotted outline indicates gene requires more data to confirm its identity. |

| 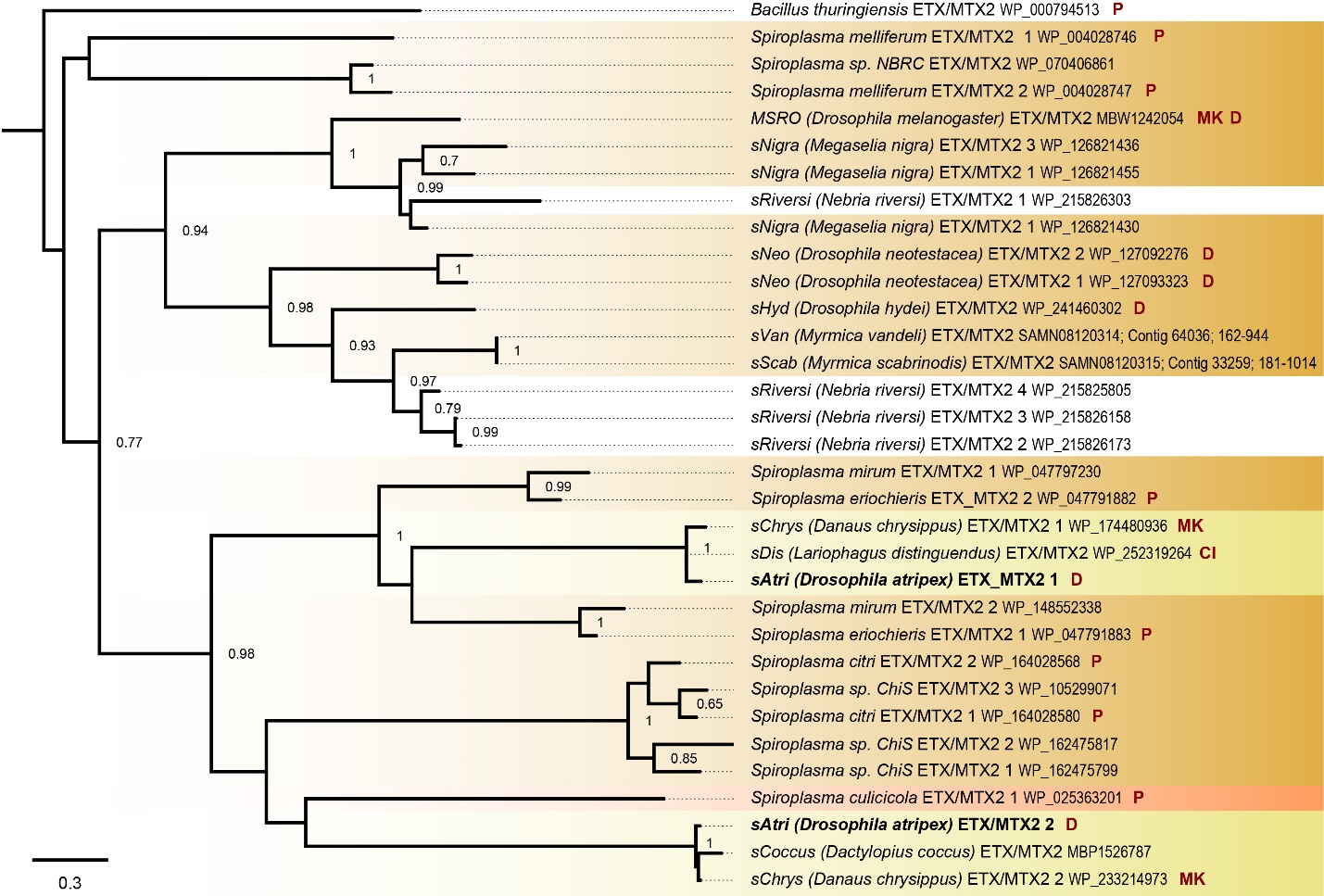 |
| --- |
|  |
| **Figure S3. *Spiroplasma* ETX/MTX2 Phylogeny.** WAG+G+F PhyML substitution model phylogeny built from MAFFT alignment of *Spiroplasma* ETX/MTX2 proteins and rooted to *Bacillus thuringiensis* ETX/MTX2. ETX/MTX2 toxin’s evolutionary history includes gene duplications and horizontal gene transfers. ETX/MTX2 domains have a notable presence among pathogenic *Spiroplasma* compared to other domains reviewed in this study. Red shading indicates Apis clade, orange shading indicates Citri clade and yellow shading indicates Ixodetis clade. Abbreviations indicate known phenotypes of the strain from which each domain was extracted (P: pathogenic, MK: male-killing, CI: cytoplasmic incompatibility-inducing, D: defensive). *sAtri* ETX/MXT2 proteins are highlighted in bold. Support values above .60 are shown. |
| **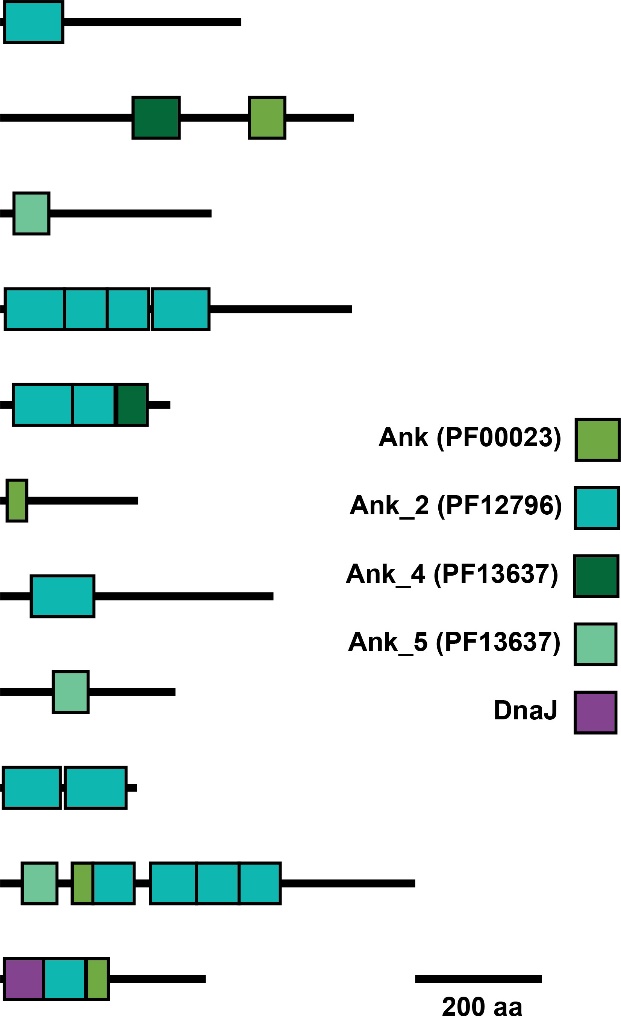** |
| **Figure S4. Diverse architectures of *sAtri* ankyrin proteins.** The *sAtri* genome encodes eleven ankyrin domain-possessing proteins, one of which can be found on a plasmid. These domains are especially numerous across the Ixodetis clade compared to Citri clade members. |

| 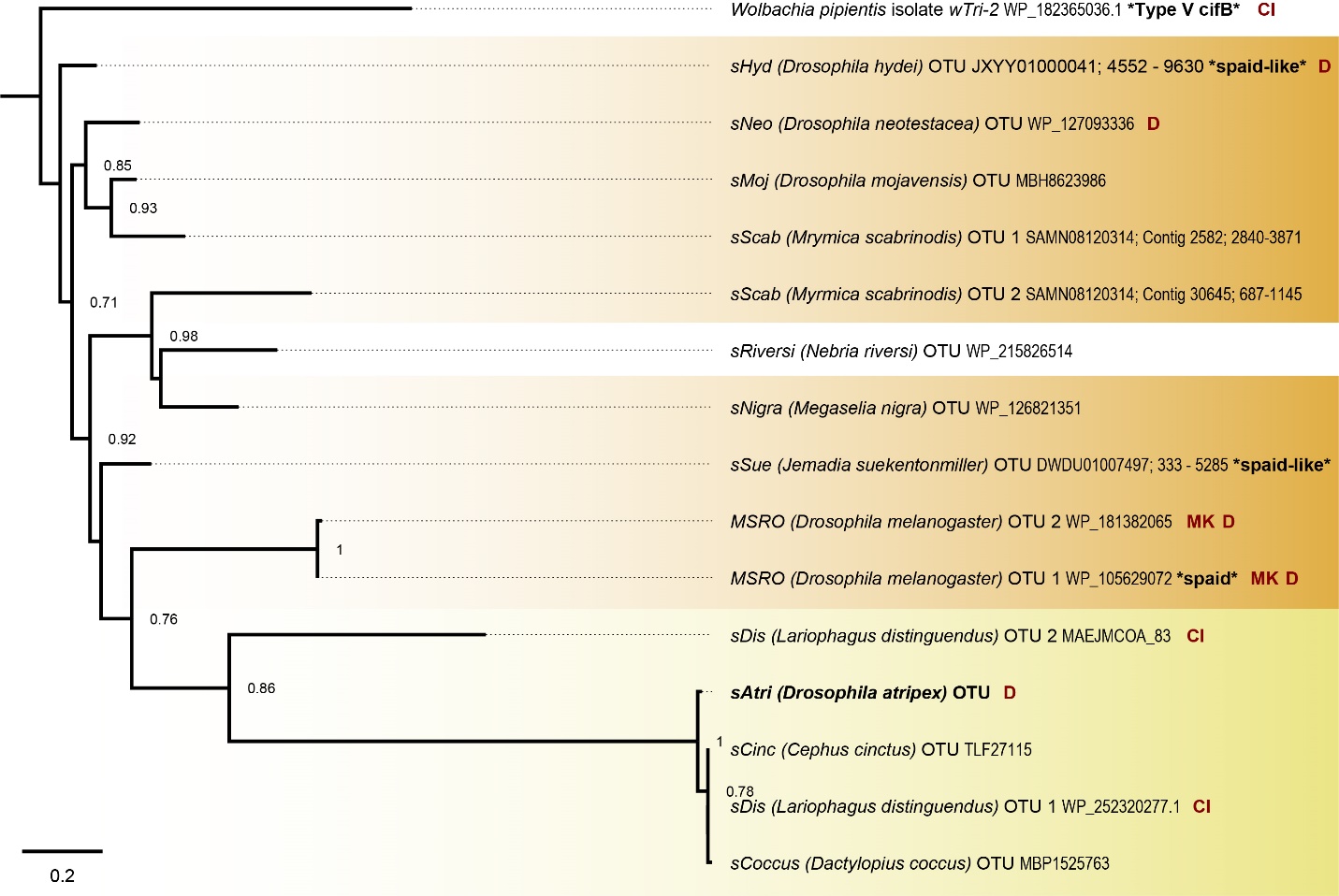 |
| --- |
| **Figure S5.** ***Spiroplasma* OTU Phylogeny.** WAG+G+F PhyML substitution model phylogeny built from MAFFT alignment of *Spiroplasma* of out and rooted to *Wolbachia pipientis* OTU. Domains show conserved evolution with Ixodetis and Citri OTU’s grouping among their respective clades. Type V cifB OTU from *Wolbachia pipientis* is used as an outgroup for reference. Orange shading indicates Citri clade and yellow shading indicates Ixodetis clade. OTU domains extracted from the male-killing spaid toxin and the CI-like cifB toxin are noted. Abbreviations indicate known phenotypes of the strain from which each domain was extracted: (MK: male-killing, CI: cytoplasmic incompatibility-inducing, D: defensive). *sAtri* OTU is highlighted in bold. Support values above .60 are shown. |
| 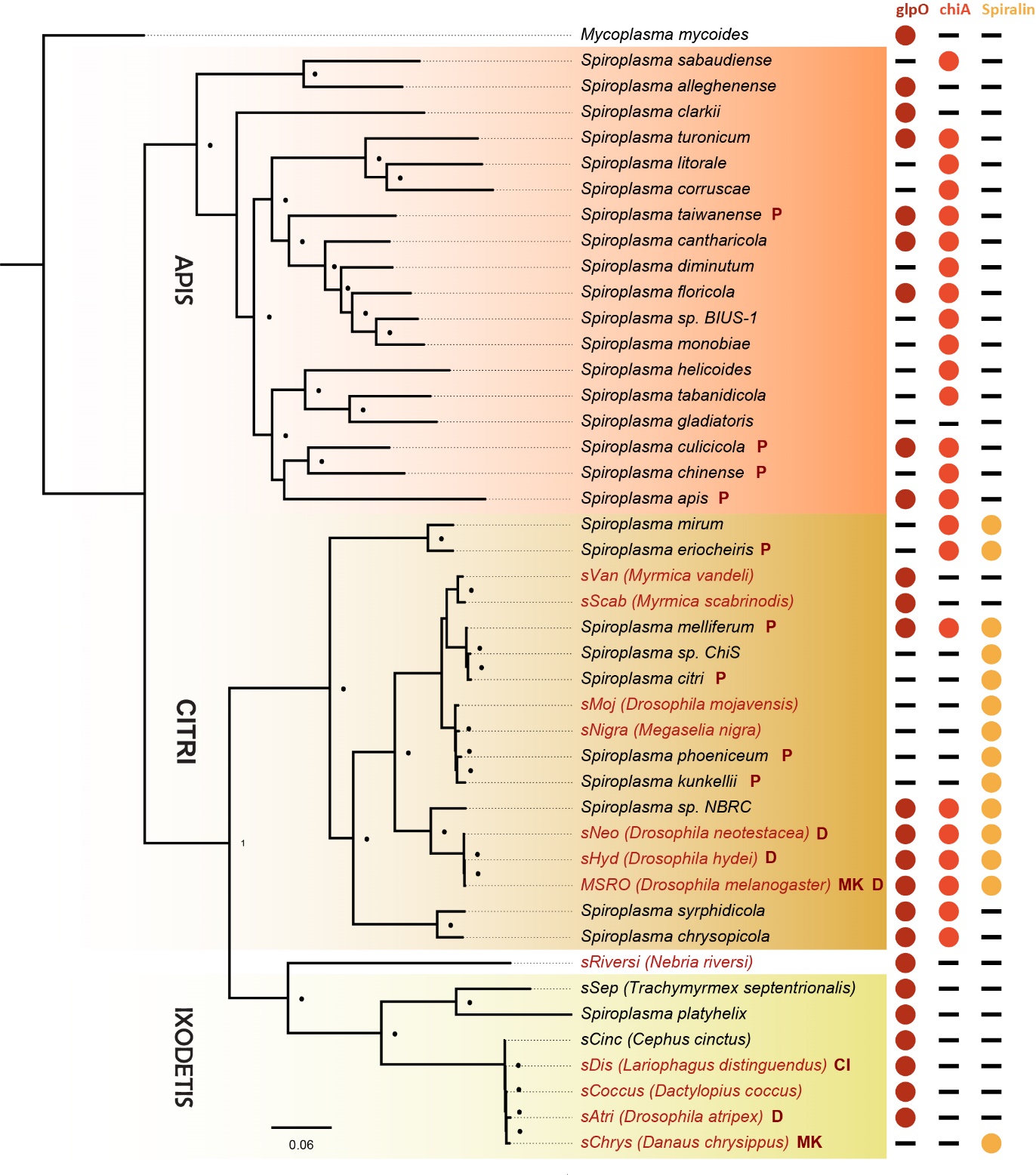 |
| **Figure S6. Phylogenetic distribution of putative virulence domains in the genus Spiroplasma.** FastTree phylogeny built from MAFFT alignments of concatenated *Spiroplasma* ftsZ, rpoB and gyrB and rooted to *M. mycoides*. glpO, chiA and Spiralin domains show little or no distribution correlated with a heritable lifestyle. Small black dots next to nodes indicate FreeTree support values greater than 75%. Large, colored dots to the right of branch labels indicates at least one domain copy present in the genome. Black dash indicates no domain copies present in the genome. Final column shows number of phage regions present in the genome as determined by Phaster. Red text is used for VT *Spiroplasma* and black text is used for non-VT *Spiroplasma*. Abbreviations indicate known phenotypes of the strain from which each domain was extracted: (P: pathogenic, MK: male-killing, CI: cytoplasmic incompatibility-inducing, D: defensive). |
|  |

| 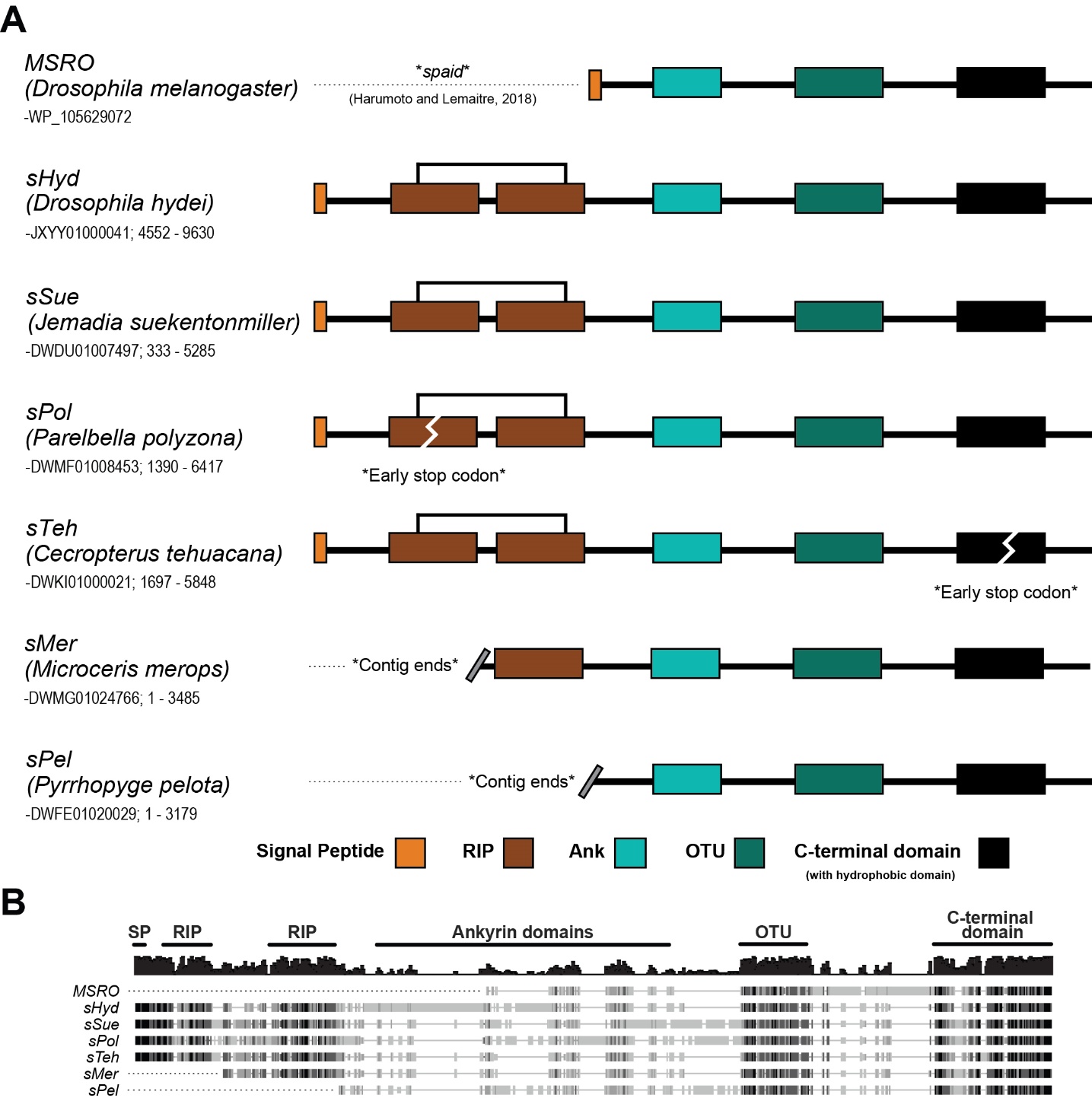 |
| --- |
| **Figure S7. *spaid­*-like toxins are present across *Spiroplasma*.** A) An unscaled representation of Spaid-like toxins distributed across *Spiroplasma*. These toxins are especially widespread in *Spiroplasma* infecting hesperid butterflies. A bracket is used to highlight that the two separated RIP domains make up a whole RIP domain. The presence of a Spaid-like C-terminal domain was determined by alignments and the presence of a hydrophobic domain. B) MAFFT alignment of Spaid and Spaid-like proteins showing domain-dependent levels of amino acid conservation. Particularly the ankyrin domain region and the region between the two RIP domains are subject to higher rates of evolution compared to other domains. |
