# Supplementary material for: The toxins of vertically transmitted Spiroplasma
Source: Front Microbiol. 2023 May 18;14:1148263. doi: 10.3389/fmicb.2023.1148263 (PMC10232968; doi:10.3389/fmicb.2023.1148263)
Supplement: Supplementary file 1 [file Data_Sheet_1.zip › Supplementary Tables S1 S4 and S5.DOCX]

| **Supplementary tables 1, 4 and 5**   \| **Table S1. sAtri genome long read coverage depth** \| \| \| \| \| --- \| --- \| --- \| --- \| \| **ID** \| **Type** \| **Length (bp)** \| **Hybrid assembly**†* \| \| PPFGHCPK_1 \| Chromosome \| 1,271,056 \| 1 \| \| PPFGHCPK_2 \| Unplaced contig†† \| 42,797 \| 4.79 \| \| PPFGHCPK_3 \| Plasmid \| 22,183 \| 6.74 \| \| PPFGHCPK_4 \| Plasmid \| 19,639 \| 6.29 \| \| PPFGHCPK_5 \| Plasmid \| 19,212 \| 9.38 \| \| PPFGHCPK_6 \| Plasmid \| 18,854 \| 6.57 \| \| PPFGHCPK_7 \| Plasmid \| 18,402 \| 6.5 \| \| PPFGHCPK_8 \| Plasmid \| 18,250 \| 6.33 \| \| PPFGHCPK_9 \| Plasmid \| 11,008 \| 8.21 \| \| †Assembled with Unicycler using 10 kb+ read length \| \| \| \| \| *Unicycler contig coverage is normalized against chromosome coverage \| \| \| \| \| ††Contig did not close during assembly \| \| \| \|   **Table S4. WGS-extracted *Spiroplasma* genome statistics** | | | | | | | | | |
| --- | --- | --- | --- | --- | --- | --- | --- | --- | --- | --- | --- | --- | --- | --- | --- | --- | --- | --- | --- | --- | --- | --- | --- | --- | --- | --- | --- | --- | --- | --- | --- | --- | --- | --- | --- | --- | --- | --- | --- | --- | --- | --- | --- | --- | --- | --- | --- | --- | --- | --- | --- | --- | --- | --- | --- | --- | --- | --- | --- | --- | --- | --- | --- | --- | --- |
| **Host** | **Abb.** | **WGS Accession** | **Size** | **Contigs** | **%GC** | **N50** | **CDS** | **tRNA** | **(%)**  **Complete** |
| *Microceris merops* | *sMer* | GCA_018244855.1 | 1,669,734 | 442 | 24.5 | 8781 | 1777 | 34 | 96.69 |
| *Cecropterus tehuacana* | *sTeh* | GCA_018249375.1 | 1,542,816 | 1117 | 26.8 | 2840 | 1151 | 16 | 92.72 |
| *Elbella theseus* | *sThe* | GCA_018245175.1 | 1,082,031 | 609 | 26.5 | 2368 | 1064 | 10 | 44.37 |
| *Parelbella polyzona* | *sPol* | GCA_018244815.1 | 1,015,402 | 244 | 26.6 | 6566 | 1083 | 28 | 97.35 |
| *Microceris iphinous* | *sIph* | GCA_018246495.1 | 955,647 | 596 | 26.8 | 2129 | 863 | 10 | 62.91 |
| *Cecropterus toxeus* | *sTox* | GCA_018249875.1 | 991,304 | 215 | 26.8 | 7898 | 1015 | 20 | 96.69 |
| *Jemadia suekentonmiller* | *sSue* | GCA_018245575.1 | 1,136,333 | 336 | 26.1 | 6811 | 1173 | 33 | 96.69 |
| *Pyrrhopyge hadassa* | *sHad* | GCA_018251255.1 | 1,310,673 | 1078 | 26.6 | 1431 | 1010 | 21 | 37.09 |
| *Monomorium pharaonis* | *sPharaoh* | GCA_003575265.1 | 780,137 | 31 | 30.9 | 45009 | 727 | 28 | 98.01 |
| *Pogonognathellus longicornis* | *sLong* | GCA_019775785.1 | 1,235,120 | 32 | 27.3 | 99815 | 1084 | 28 | 97.35 |
| *Formica Selysi* | *sSelysi* | GCA_009859135.1 | 1,229,451 | 4 | 23.8 | 603476 | 1545 | 29 | 94.04 |
| *Colias croceus* | *sCroceus* | GCA_009982905.1 | 1,902,601 | 51 | 22.8 | 100046 | 2492 | 28 | 97.35 |
| *Zaprionus kolodkinae* | *sKolod* | GCA_018901885.1 | 981,540 | 35 | 23.6 | 81665 | 1175 | 11 | 74.83 |

| **Table S5 *Spiroplasma* abbreviations and accession numbers** | | |
| --- | --- | --- |
| **Apis *Spiroplasma*** | **Abbreviation** | **Accession** |
| *Spiroplasma floricola* | - | [NZ_CP025057.1](https://www.ncbi.nlm.nih.gov/nuccore/NZ_CP025057.1) |
| *Spiroplasma monobiae* | - | [NZ_CP025543.1](https://www.ncbi.nlm.nih.gov/nuccore/NZ_CP025543.1) |
| *Spiroplasma apis* | - | [NC_022998.1](https://www.ncbi.nlm.nih.gov/nuccore/NC_022998.1) |
| *Spiroplasma sabaudiense* | - | [NZ_CP006934.1](https://www.ncbi.nlm.nih.gov/nuccore/NZ_CP006934.1) |
| *Spiroplasma culicicola* | - | [NZ_CP006681.1](https://www.ncbi.nlm.nih.gov/nuccore/NZ_CP006681.1) |
| *Spiroplasma taiwanense* | - | [GCA_000439435.1](https://www.ncbi.nlm.nih.gov/assembly/45551) |
| *Spiroplasma diminutum* | - | [NC_021833.1](https://www.ncbi.nlm.nih.gov/nuccore/NC_021833.1) |
| *Spiroplasma turonicum* | - | [NZ_CP012328.1](https://www.ncbi.nlm.nih.gov/nuccore/NZ_CP012328.1) |
| *Spiroplasma clarkii* | - | [GCA_002795265.1](https://www.ncbi.nlm.nih.gov/assembly/GCA_002795265.1) |
| *Spiroplasma alleghenense* | - | [NZ_CP031376.1](https://www.ncbi.nlm.nih.gov/nuccore/NZ_CP031376.1) |
| *Spiroplasma tabinadicola* | - | [NZ_CP046276.1](https://www.ncbi.nlm.nih.gov/nuccore/NZ_CP046276.1) |
| *Spiroplasma chinense* | - | [NZ_CP043026.1](https://www.ncbi.nlm.nih.gov/nuccore/NZ_CP043026.1) |
| *Spiroplasma gladiatoris* | - | [NZ_CP038013.1](https://www.ncbi.nlm.nih.gov/nuccore/NZ_CP038013.1) |
| *Spiroplasma corruscae* | - | [GCA_002237575.1](https://www.ncbi.nlm.nih.gov/assembly/1163171) |
| *Spiroplasma helicoides* | - | [NZ_CP017015.1](https://www.ncbi.nlm.nih.gov/nuccore/NZ_CP017015.1) |
| *Spiroplasma cantharicola* | - | [NZ_CP012622.1](https://www.ncbi.nlm.nih.gov/nuccore/NZ_CP012622.1) |
| *Spiroplasma litorale* | - | [NZ_CP012357.1](https://www.ncbi.nlm.nih.gov/nuccore/NZ_CP012357.1) |
| *Spiroplasma sp. BIUS-1* | - | GCA_010365805.1 |
| **Citri *Spiroplasma*** | **Abbreviation** | **Accession** |
| *Spiroplasma* of *Drosophila neotestacea* | *sNeo* | [GCA_003989055.1](https://www.ncbi.nlm.nih.gov/assembly/GCA_003989055.1) |
| *Spiroplasma* of *Drosophila melanogaster* | *MSRO* | [GCA_000820525.2](https://www.ncbi.nlm.nih.gov/assembly/GCA_000820525.2) |
| *Spiroplasma* of *Drosophila hydei* | *sHyd* | GCA_016082235.1 |
| *Spiroplasma* of *Drosophila mojavensis* | *sMoj* | [GCA_016082285.1](https://www.ncbi.nlm.nih.gov/assembly/GCA_016082285.1) |
| *Spiroplasma* of *Megaselia nigra* | *sNigra* | [GCA_003987485.1](https://www.ncbi.nlm.nih.gov/assembly/GCA_003987485.1) |
| *Spiroplasma* of *Myrmica scabrinodis* | *sScab* | SAMN08120315 |
| *Spiroplasma* of *Myrmica vandeli* | *sVan* | SAMN08120314 |
| *Spiroplasma sp. ChiS* | - | [GCA_002968355.1](https://www.ncbi.nlm.nih.gov/assembly/GCA_002968355.1) |
| *Spiroplasma sp. NBRC* | - | [GCA_001886495.1](https://www.ncbi.nlm.nih.gov/assembly/GCA_001886495.1) |
| *Spiroplasma melliferum* | - | [GCA_000236085.3](https://www.ncbi.nlm.nih.gov/assembly/GCA_000236085.3) |
| *Spiroplasma mirum* | - | [NZ_CP002082.1](https://www.ncbi.nlm.nih.gov/nuccore/NZ_CP002082.1) |
| *Spiroplasma eriocheiris* | - | [NZ_CP011856.1](https://www.ncbi.nlm.nih.gov/nuccore/NZ_CP011856.1) |
| *Spiroplasma phoeniceum* | - | [GCA_003339775.1](https://www.ncbi.nlm.nih.gov/assembly/1834121) |
| *Spiroplasma syrphidicola* | - | [NC_021284.1](https://www.ncbi.nlm.nih.gov/nuccore/NC_021284.1) |
| *Spiroplasma chrysopicola* | - | [NC_021280.1](https://www.ncbi.nlm.nih.gov/nuccore/NC_021280.1) |
| *Spiroplasma kunkelii* | - | [GCA_001274875.1](https://www.ncbi.nlm.nih.gov/assembly/476501) |
| *Spiroplasma citri* | - | [GCA_013113755.1](https://www.ncbi.nlm.nih.gov/assembly/GCA_013113755.1) |
| **Ixodetis *Spiroplasma*** | **Abbreviation** | **Accession** |
| *Spiroplasma* of *Dactylopius coccus* | *sCoccus* | [GCA_017847635.1](https://www.ncbi.nlm.nih.gov/assembly/GCA_017847635.1) |
| *Spiroplasma* of *Danaus chrysippus* | *sChrys* | [GCA_902809815.1](https://www.ncbi.nlm.nih.gov/assembly/GCA_902809815.1) |
| *Spiroplasma* of *Lariophagus distinguendus* | *sDis* | GCA_023846195.1 |
| *Spiroplasma* of *Cephus cinctus* | *sCinc* | [GCA_005795965.1](https://www.ncbi.nlm.nih.gov/assembly/GCA_005795965.1) |
| *Spiroplasma* of *Drosophila atripex* | *sAtri* | - |
| *Spiroplasma platyhelix* | - | [GCA_012163225.1](https://www.ncbi.nlm.nih.gov/assembly/GCA_012163225.1) |
| *Spiroplasma* of *Trachymyrmex septentrionalis* | *sSep* | GCA_023509825.1 |
| **Unassigned *Spiroplasma*** | **Abbreviation** | **Accession** |
| *Spiroplasma* of *Nebria riversi* | *Riversi* | [GCA_018831625.1](https://www.ncbi.nlm.nih.gov/assembly/GCA_018831625.1) |
| Plasmids, if present in a *Spiroplasma sp.*, are included in the GCA_* accession download | | |
